# MH-76, a Novel Non-Quinazoline α_1_-Adrenoceptor Antagonist, but Not Prazosin Reduces Inflammation and Improves Insulin Signaling in Adipose Tissue of Fructose-Fed Rats

**DOI:** 10.3390/ph14050477

**Published:** 2021-05-18

**Authors:** Monika Kubacka, Szczepan Mogilski, Monika Zadrożna, Barbara Nowak, Małgorzata Szafarz, Bartosz Pomierny, Henryk Marona, Anna Waszkielewicz, Wojciech Jawień, Jacek Sapa, Marek Bednarski, Joanna Knutelska, Magdalena Kotańska

**Affiliations:** 1 Department of Pharmacodynamics, Faculty of Pharmacy, Medical College, Jagiellonian University, Medyczna 9, 30-688 Kraków, Poland; szczepan.mogilski@uj.edu.pl (S.M.); jacek.sapa@uj.edu.pl (J.S.); 2 Department of Cytobiology, Faculty of Pharmacy, Medical College, Jagiellonian University, Medyczna 9, 30-688 Kraków, Poland; monika.zadrozna@uj.edu.pl (M.Z.); barbara.anna.nowak@uj.edu.pl (B.N.); 3 Department of Pharmacokinetics and Physical Pharmacy, Faculty of Pharmacy, Medical College, Jagiellonian University, Medyczna 9, 30-688 Kraków, Poland; malgorzata.szafarz@uj.edu.pl; 4 Department of Biochemical Toxicology, Medical College, Jagiellonian University, Medyczna 9, 30-688 Kraków, Poland; bartosz.pomierny@uj.edu.pl; 5 Department of Bioorganic Chemistry, Chair of Organic Chemistry, Faculty of Pharmacy, Medical College, Jagiellonian University, Medyczna 9, 30-688 Kraków, Poland; henryk.marona@uj.edu.pl (H.M.); anna.waszkielewicz@uj.edu.pl (A.W.); 6 Department of Pharmaceutical Biophysics, Faculty of Pharmacy, Medical College, Jagiellonian University, Medyczna 9, 30-688 Kraków, Poland; wojciech.jawien@uj.edu.pl; 7 Department of Pharmacological Screening, Faculty of Pharmacy, Jagiellonian University, Medical College, 9 Medyczna Street, 30-688 Kraków, Poland; marek.bednarski@uj.edu.pl (M.B.); joanna.knuteiska@uj.edu.pl (J.K.); magda.dudek@uj.edu.pl (M.K.)

**Keywords:** adipose tissue, α1-adrenoceptors antagonist, inflammation, adipocytokines, insulin resistance, obesity, metabolic syndrome, fructose-fed rats

## Abstract

Background: Quinazoline α_1_-adrenoceptors antagonists have been shown to exert moderately favorable effects on the metabolic profile in hypertensive patients. However, based on AntiHypertensive and Lipid-Lowering Treatment to Prevent Heart Attack Trial (ALLHAT) results, they are no longer recommended as a first line therapy of hypertension. Recent studies have shown that quinazoline-based α_1_-adrenoceptors antagonists (prazosin, doxazosin) induce the apoptosis and necrosis, which may be responsible for ALLHAT outcomes; however, these effects were proven to be independent of α_1_-adrenoceptor blockade and were associated with the presence of quinazoline moiety. MH-76 (1-[3-(2,6-dimethylphenoxy)propyl]-4-(2-methoxyphenyl)piperazine hydrochloride)) is a non-quinazoline α_1_-adrenoceptor antagonist which, in fructose-fed rats, exerted antihypertensive effect, and, contrary to prazosin, reduced insulin resistance and abdominal adiposity. In this study we aimed to further investigate and compare the effects of MH-76 and prazosin on inflammation in adipose tissue of fructose-fed rats. Methods: Abdominal adipose tissue was collected from four groups of fructose-fed rats (Control, Fructose, Fructose + MH-76 and Fructose + Prazosin) and subjected to biochemical, histopathological and immunohistochemical studies. Moreover, selected tissue distribution studies were performed. Results: Treatment with MH-76 but not with prazosin improved endothelial integrity, reduced adipose tissue inflammation and infiltration by immune cells, resulting in lowering leptin, MCP-1, IL-6, TNF-α and PAI-1 levels. In adipose tissue from Fructose + MH-76 animals, a higher amount of eosinophils accompanied with higher IL-4 concentration was observed. Treatment with MH-76 but not with prazosin markedly reduced phosphorylation of IRS-1 at Ser307. Conclusion: MH-76 may improve insulin signaling in adipose tissue by reducing the pro-inflammatory cytokine production and inhibiting the inflammatory cells recruitment. In contrast, in adipose tissue from animals treated with prazosin, the inflammatory effect was clearly enhanced.

## 1. Introduction

Metabolic syndrome, an epidemic disorder of a global nature, is a cluster of risk factors (i.e., abdominal adiposity, dyslipidemia, high blood pressure, insulin resistance and proinflammatory states) that increase the likelihood of developing diabetes mellitus and cardiovascular disorders. Excessive fructose consumption has been associated with the pathogenesis of metabolic syndrome, as it leads to abdominal obesity and intrahepatic fat accumulation, which in turn may evolve into the non-alcoholic fatty liver disease (NAFLD), a hepatic manifestation of metabolic syndrome. In fact, abdominal obesity is regarded as the major cause of insulin resistance [1,2,3,4,5,6]. Adipose tissue releases free fatty acids that contribute to the development of insulin resistance in liver and muscles. Moreover, it is responsible for the persistent inflammation subsequently augmenting insulin resistance. Additionally, low-grade chronic inflammation affects local adipose physiology and exerts systemic detrimental effects on other tissues [1,4,7]. This is primarily due to macrophage infiltration as well as increased secretion of a large number of pro-inflammatory and decreased secretion of anti-inflammatory substances, like adipokines (adiponectin, resistin), hormones (leptin) and cyto- and chemokines (i.e., monocyte chemoattractant protein 1 (MCP-1), tumor necrosis factor α (TNF-α) and interleukin-6 (IL-6) [3,4,8,9,10,11]. This inflammatory condition may alter the insulin signaling cascade in adipose tissue that includes insulin receptor substrate 1 (IRS-1) phosphorylation at the serine307 residue and, consequently, the decrease of protein kinase B (PKB/Akt) activity and Glucose transporter type 4 (GLUT-4) translocation and glucose transport [12].

Obesity and insulin resistance together with compensatory hyperinsulinemia exert sympatho-stimulatory effect, sympathetic over-activity and α_1_-adrenoceptor activation, which may lead to vasoconstriction and play a role, among others factors, in pathogenesis of hypertension [3,7,13]. Quinazoline-based α_1_-adrenoceptors antagonists such as prazosin or doxazosin have been used for years in the treatment of hypertension and they have been shown, in addition to their antihypertensive effect, to moderately improve the metabolic profile of the hypertensive patients, mainly through vasodilatation of blood vessels and improvement of regional blood flow and tissue glucose delivery [14,15,16]. However, in the Antihypertensive and Lipid-Lowering Treatment to Prevent Heart Attack Trial (ALLHAT) study, doxazosin was associated with higher risk of combined cardiovascular disease events, and since then α_1_-adrenoceptors antagonists are no longer recommended as a first line therapy of hypertension [13,17]. Recent studies have shown that quinazoline-based α_1_-adrenoceptors antagonists induce the apoptosis and necrosis of cardiomyocytes and other cell types, which may be responsible, at least in part, for ALLHAT outcomes; however, apoptosis induced by quinazoline-based α_1_-adrenergic antagonists was proven to be independent of α_1_-adrenoceptor blockade and is associated with the presence of quinazoline moiety. Moreover, apoptosis induction was never shown for non-quinazoline-based α_1_- adrenoceptors antagonists such as tamsulosin or urapidil [17,18,19,20,21].

**Figure 1:**
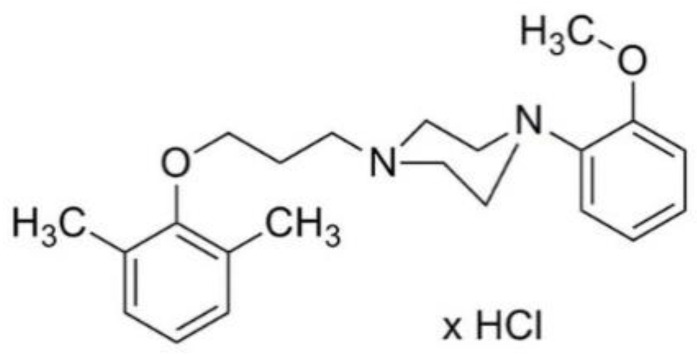
Structure of MH-76.

MH-76 (1-[3-(2,6-dimethylphenoxy)propyl]-4-(2-methoxyphenyl)piperazine hydrochloride) (Figure 1) does not contain quinazoline moiety in its structure and may be regarded as urapidil analogue. It is a reversible and competitive antagonist of α_1_-adrenoceptors, with no selectivity for a specific α_1_-adrenoceptor subtype. We have showed previously that MH-76 has a potent hypotensive activity [22]. Moreover, it exerts some additional pleiotropic features, such as activation of the endothelial NO—cGMP signaling pathway [23].

In our previous study we showed that MH-76, in fructose-fed rats with metabolic syndrome, exerted antihypertensive effect, reversed endothelial dysfunction, decreased hyperglycemia and hypertriglyceridemia and reduced insulin resistance and abdominal adiposity. Moreover, MH-76 decreased TNF-α concentration and lipid peroxidation in adipose tissue. Prazosin treatment also exerted an antihypertensive effect and reduced hyperglycemia; however, it had no other beneficial effects characteristic for MH-76 such as improvement of endothelial dysfunction, reduction of insulin resistance and the abdominal adiposity [24].

The present study was conducted to compare the effects of MH-76 and prazosin on some of the critical points associated with pro-inflammatory state and insulin signaling and their ability to affect adipocytokines in adipose tissue of fructose-fed rats, a well validated model of metabolic syndrome [25,26].

## 2. Results

### 2.1. In Vitro Functional Bioassays on Cells Transfected with Human Adrenergic α_1A_-Receptor

The intrinsic activity of MH-76 for α_1A_ subtype of adrenergic receptor was examined using surefire method. MH-76 was tested for both the agonistic and antagonistic activities by aequorin-based functional assay. It did not show any agonistic activity; however, proved to be the strong α_1A_-adrenergic receptor antagonist with the IC_50_ value equal to 1.42 nM, whereas IC_50_ value for prazosin was about 0.68 nM. The results are summarized in Table 1.

### 2.2. Effect of MH-76 and Prazosin on the Concentration of Pro- and Anti-Inflammatory Adipocytokines in Abdominal Adipose Tissue

**Figure 2:**
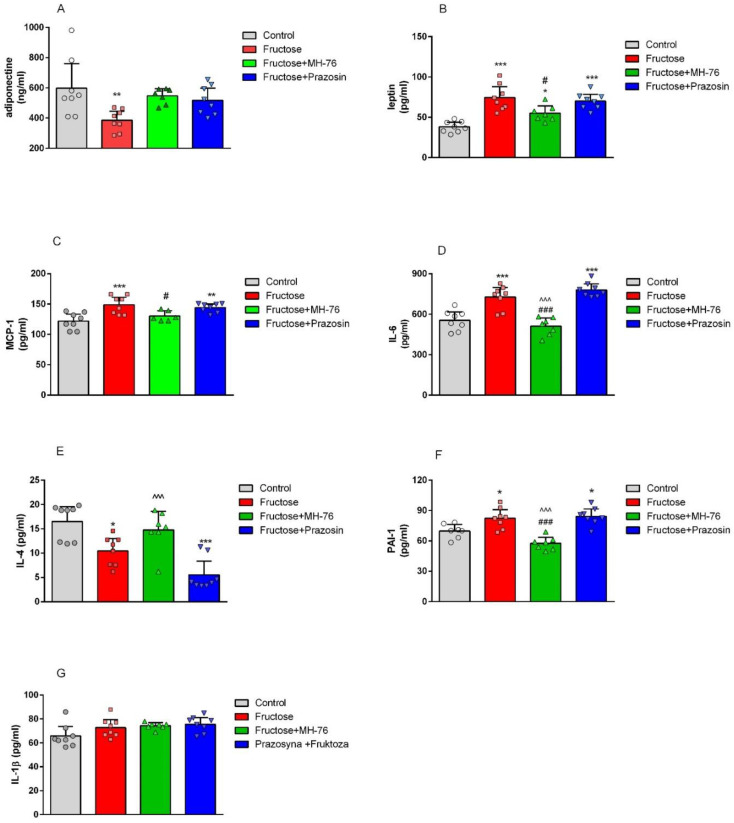
Effects of MH-76 (5 mg/kg i.p.) and prazosin (0.2 mg/kg i.p.) on adiponectin (**A**), leptin (**B**), MCP-1 (**C**), IL-6 (**D**), IL-4 (**E**), PAI-1 (**F**) and IL-1β (**G**) concentration in fructose-fed rats adipose tissue. Data are expressed as means ± Δ/2, where Δ is a width of the 95% confidence interval (CI), (n = 6–8), * *p* < 0.05, ** *p* < 0.01, *** *p* < 0.001 vs. Control, # *p* < 0.05, ### *p* < 0.001 vs. Fructose, ^^^ *p* < 0.001 vs. Fructose + Prazosin group (one-way ANOVA, post hoc Bonferroni multiple comparisons test).

As shown in Figure 2A, fructose consumption resulted in decreased adiponectine (by 35.45%, *p* < 0.01) and increased leptin (by 95.58%, *p* < 0.001, Figure 2B) levels in rats’ adipose tissue. Treatment with both α_1_- adrenoceptors antagonists restored the concentration of adiponectine in adipose tissue (Figure 2A). However, treatment with MH-76 but not with prazosin decreased the elevated leptin level (74.38 ± 5.68 vs. 55.00 ± 3.68 pg/mL, *p* < 0.05) (Figure 2B).

As shown in Figure 2C,D, fructose feeding caused a statistically significant increase in MCP-1 (by 21.81%, *p* < 0.001) and IL-6 (by 31.02%, *p* < 0.001) concentrations in abdominal adipose tissue, compared with the values observed in rats on the standard diet. Treatment with MH-76 normalized the elevated MCP-1 and IL-6 concentrations, whereas prazosin was not effective.

Excessive fructose consumption resulted in lowering interleukin-4 (IL-4) concentration (by 36.77%, *p* < 0.05) in adipose tissue, as shown in Figure 2E. Treatment with MH-76, contrary to prazosin tended to increase the decreased IL-4 levels.

Fructose consumption led to an increase in PAI-1 concentration compared to control (Control—100% vs. Fructose—118.14%, *p* < 0.05, Figure 2F). Treatment with MH-76 but not with prazosin effectively lowered PAI-1 level (Fructose—100% vs. Fructose + MH-76—69.89%, *p* < 0.001, Figure 2F).

There was no statistically significant difference in interleukin-1β (IL-1β) concentration in abdominal adipose tissue among experimental groups (Figure 2G).

### 2.3. Effect of MH-76 and Prazosin on Insulin Signaling in Abdominal Adipose Tissue

**Figure 3:**
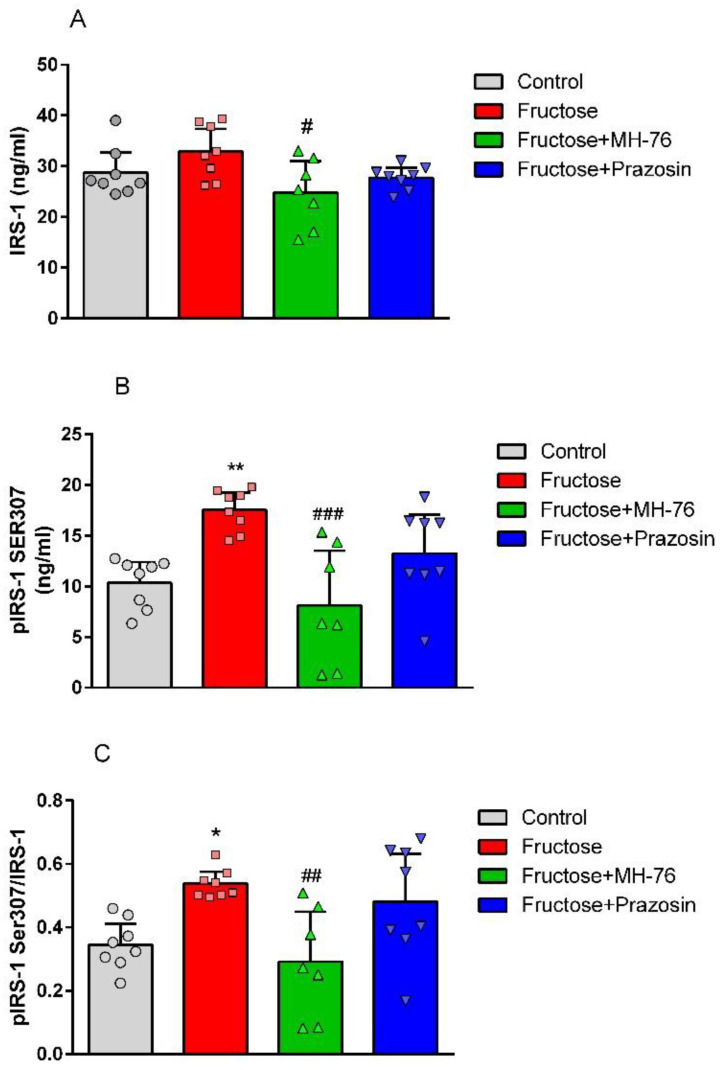
Effects of MH-76 (5 mg/kg i.p.) and prazosin (0.2 mg/kg i.p.) on (**A**) IRS-1 and (**B**) pIRS-1 Ser307 concentration in fructose-fed rats adipose tissue. (**C**) Ratio of pIRS-1 Ser307 to IRS-1 concentration in different experimental groups. Data are expressed as means ± Δ/2, where Δ is a width of the 95% CI, (n = 7–8), * *p* < 0.05, ** *p* < 0.01 vs. Control, # *p* < 0.05, ## *p* < 0.01, ### *p* < 0.001 vs. Fructose group (one-way ANOVA, post hoc Bonferroni multiple comparisons test).

The high fructose diet induced a statistically significant (*p* < 0.01) phosphorylation of IRS-1 at Ser307 (Control—100 % vs. Fructose—169.17%, Figure 3B) with no statistically significant effect on total IRS-1 concentration (Figure 3A) in abdominal adipose tissue, compared to control rats. Treatment with MH-76 decreased the concentration of the total IRS-1 (Fructose—100% vs. Fructose + MH-76—75.36%, *p* < 0.05) and reduced phosphorylation of IRS-1 at Ser307 comparing to rats fed with fructose alone (Fructose—100% vs. Fructose + MH-76—46.36%, *p* < 0.001), whereas prazosin was not effective (Figure 3B). This effect was even more pronounced when the ratios of pIRS-1Ser307 to total IRS-1 were compared (Figure 3C).

### 2.4. Histopathological Analysis of Abdominal Adipose Tissue

#### 2.4.1. Effect of MH-76 and Prazosin on Adipocytes Size

**Figure 4:**
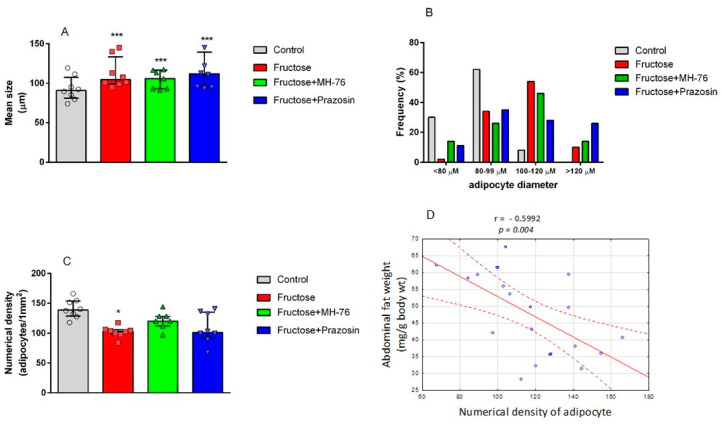
Mean size of adipocytes determined by microscopic measurement of cell diameter (**A**), profile of the distribution of adipocyte size (**B**), and numerical density of adipocytes (**C**) from abdominal adipose tissue of the Control, Fructose, Fructose + MH-76, and Fructose + Prazosin rats. Data are expressed as median + interquartile range (IQR), (**A**,**C**), * *p* < 0.05 and *** *p* < 0.001 versus Control, (Kruskal–Wallis test by ranks, post hoc Dunn test). Significant negative correlation between abdominal fat weight (mg/g body wt) and numerical density of adipocytes (Pearson correlation), (**D**).

Quantitative analyses showed a statistically significant reduction of the number of adipocytes per unit area in the Fructose group compared to the Control group (102.4 ± 5.4 vs. 140.1 ± 8.7, *p* < 0.05). Additionally we observed the tendency to increase (vs Fructose group) the numerical density of adipocytes in Fructose + MH-76 group (120.4 ± 7.8) and the values were not statistically significantly different from the Control group (Figure 4C). Moreover, there was a strong negative correlation between abdominal fat weight (mg/g body wt) and numerical density of adipocytes (Pearson correlation, r = −0.599, *p* < 0.01, Figure 4D). On the other hand, the mean size of adipocytes in all fructose-fed groups (regardless of the treatment) was similar and cells were approximately about 13% larger than those in the Control group (*p* < 0.001; Figure 4A). At the same time, the frequency distribution for each cell size diameter indicated that fructose-fed groups had higher proportion of large (100–120 µm) and very large (>120 µm) adipocytes and smaller proportion of small (<80 µm) and medium size (80–99 µm) adipocytes compared to more uniform in size fat cells localized in the Control group (Figure 4B). This combination resulted in generally bigger mean adipocyte size in fructose-fed groups. However, in the Fructose + Prazosin and Fructose + MH-76 groups we observed more small adipocytes (14% and 11%, respectively) compared to the Fructose group (2%). Moreover, the highest number of very large (>120 µm) adipocytes was observed in the Fructose + Prazosin group (26%), compared to the Fructose (10%) or Fructose + MH-76 (14%) groups.

#### 2.4.2. Effect of MH-76 and Prazosin on Inflammation in Adipose Tissue

**Figure 5:**
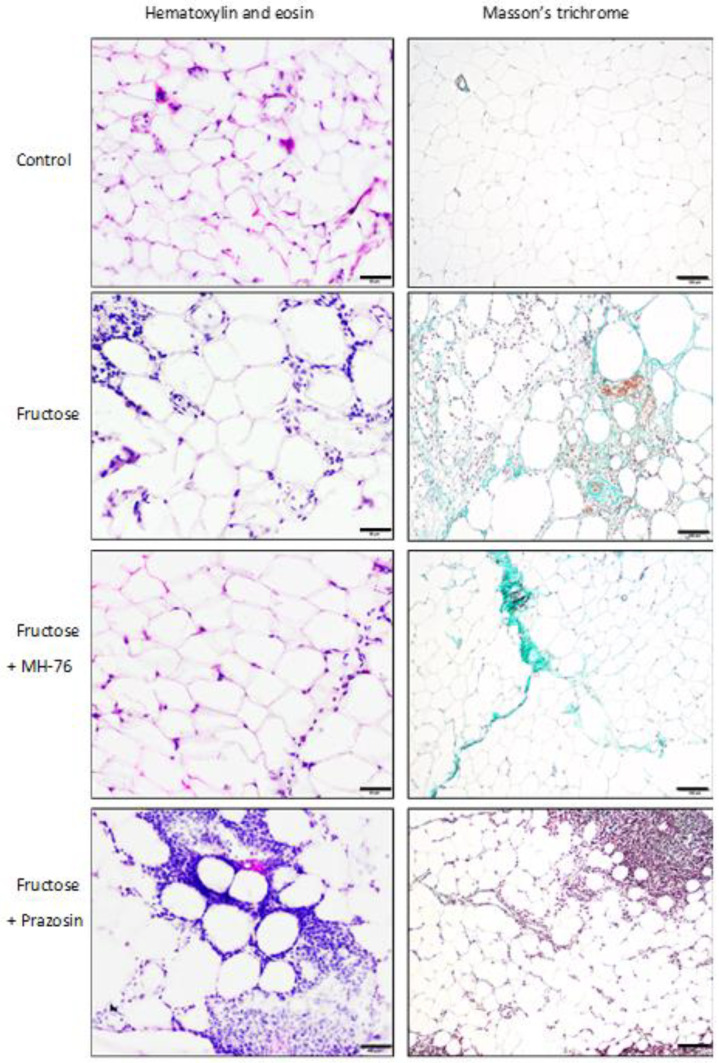
Representative histological pictures of abdominal adipose tissue sections stained with hematoxylin and eosin (bar = 20 μm) and Masson’s trichrome (bar = 100 μm). Massive amount of inflammatory cells, fibrosis and adipocyte hypertrophy are visible mainly in the Fructose and Fructose + Prazosin rats. Slight inflammation involving mostly septa are seen in the Fructose + MH-76 rats, which suggested the anti-inflammatory effect of this compound.

Histopathological examination showed a large number of mixed inflammatory cells (lymphocytes, histocytes and neutrophils) mainly in the Fructose and Fructose + Prazosin groups infiltrating the area of adipocyte occurrence—within lobules of adipose tissue. In contrast, very few inflammatory cells infiltrating mostly septa were detected in the Fructose + MH-76 group (Figure 5), while abdominal adipose tissue from the Control group showed absence of inflammatory cells. Masson’s trichrome staining of adipose tissue sections revealed that, compared with corresponding tissue material, from the fructose-fed groups, the other groups had far fewer trichrome-positive fibrotic streaks (Figure 5).

**Figure 6:**
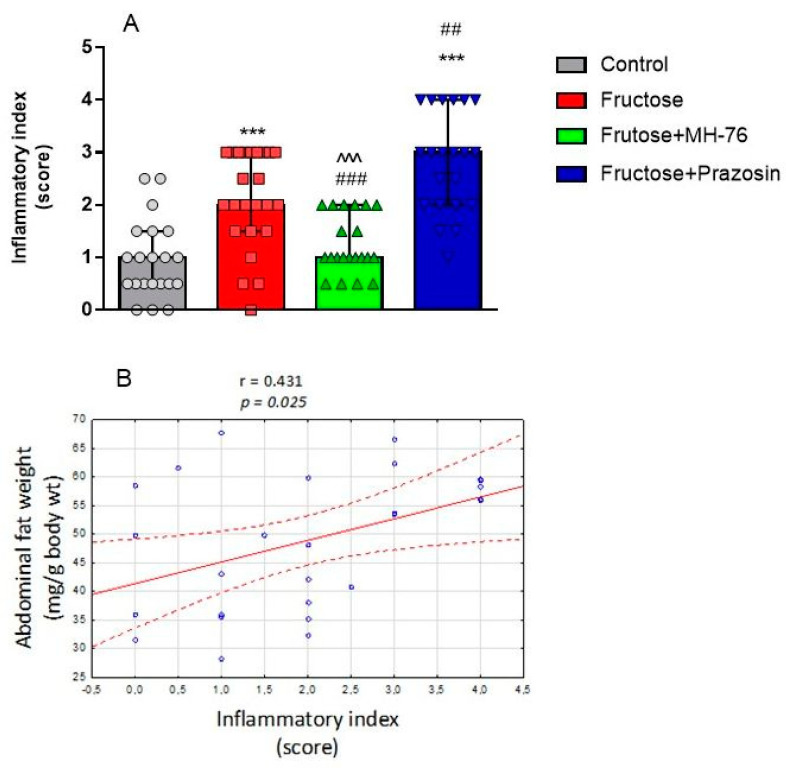
(**A**) Inflammatory index of abdominal adipose tissue in the different groups of rats. Data are expressed as median + IQR, *** *p* < 0.001 vs. Control, ## *p* < 0.01, ### *p* < 0.001 vs. Fructose, ^^^ *p* < 0.001 versus Fructose + Prazosin, (Kruskal–Wallis test by ranks, post hoc Dunn test). (**B**) Significant positive correlation between abdominal fat weight (mg/g body wt) and inflammatory index (Pearson correlation).

Thus, the abdominal adipose tissue from Fructose + MH-76 rats appears to have greater similarities with control adipose tissue than with tissue from the Fructose group with regards to inflammatory index (Figure 6A), especially that the amount of additionally observed eosinophils (Figure 9A) was also statistically significantly higher in the MH-76 treated group than in the Fructose group (*p* < 0.05; Figure 9B).

In adipose sections from the Fructose + MH-76 group we have noticed small widening of intercellular spaces, but without cellular infiltrates, which may indicate that the healing of the inflammatory process has already begun. In turn, severe inflammation in adipose tissue from the Fructose + Prazosin group was observed. There were abundant inflammatory cells infiltrating spaces between adipocytes as well as voids of various sizes formed after the breakage of the fat cells (Figure 5). The inflammatory index for the Fructose + Prazosin group was statistically significantly higher not only in comparison to Control but also to Fructose group (Figure 6A). The increase in inflammatory index correlated positively with the abdominal fat weight (mg/g body wt), (Pearson correlation, r = 0.431, *p* < 0.05, Figure 6B).

**Figure 7:**
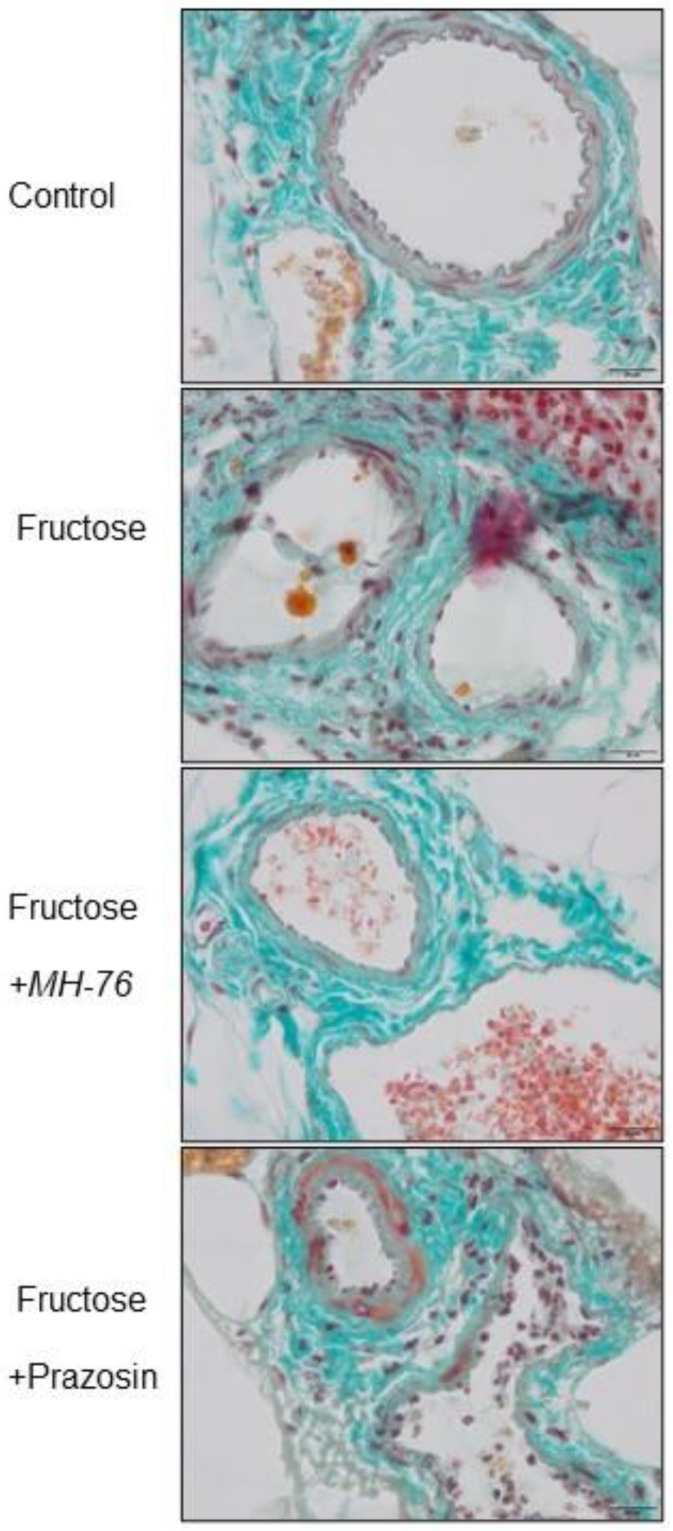
Representative histological pictures of blood microcirculation in adipose tissue sections stained with Masson’s trichrome (bar = 20 µm). The symptoms of the inflammatory phenotype (in regards to endothelial damage, leukocyte adhesion and extravasation, perivascular lymphocyte and macrophage aggregation) shown for the Fructose and Fructose + Prazosin rats.

**Figure 8:**
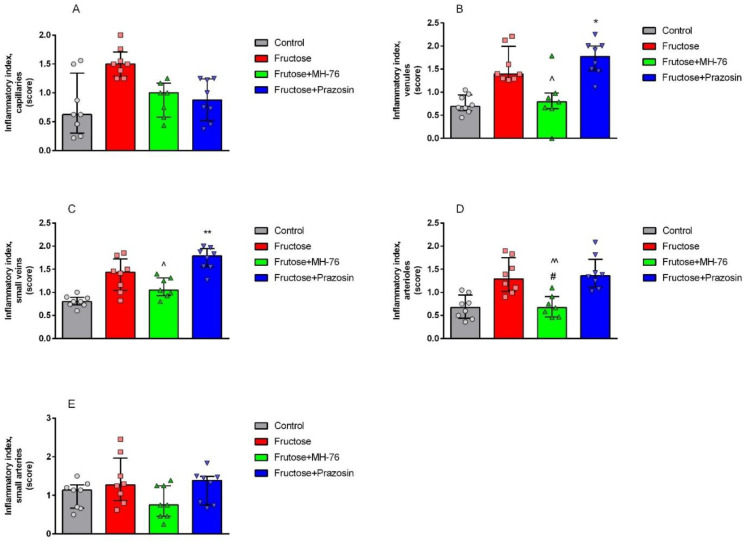
Inflammatory index of microvasculature ((**A**) capillaries, (**B**) venules, (**C**) small veins, (**D**) arterioles, (**E**) small arteries) of abdominal adipose tissue in the different groups of rats. The assessed changes included: endothelial damage, leukocyte adhesion, and extravasation, perivascular lymphocyte and macrophage aggregation. The analysis was carried out with a 40× (and 100× for endothelial damage and extravasation analyses) objective magnification, data on a 0–3 semi-quantitative scale. Separate data for the above four components were collected as a common indicator of inflammatory lesions. Data are expressed as median + IQR, * *p* < 0.05, ** *p* < 0.01 versus Control, # *p* < 0.05 versus Fructose, ^ *p* < 0.05, ^^ *p* < 0.01 versus Fructose + Prazosin, (Kruskal–Wallis test by ranks, post hoc Dunn test).

Histopathological analysis of the abdominal adipose tissue microvasculature revealed that there was the upward trend of the inflammatory symptoms (e.g., endothelial damage, leukocyte adhesion and subsequent extravasation and perivascular lymphocyte and macrophage aggregation, presented here as an averaged inflammatory index) to mild/moderate level in each microvascular component (capillaries, arterioles, and venules), and in small arteries and veins in the Fructose compared to the Control group, as shown in Figure 7 and Figure 8A–E.

Microvasculature of Fructose + MH-76 treated rats tended to display weakened, and overall mild, inflammatory symptoms comparing to the Fructose rats. For Fructose + MH-76 arterioles, frequency of signs of endothelial damage, leukocyte peripheralization and extravasation and perivascular leucocytes aggregation was statistically significantly lower than in the Fructose group (*p* < 0.05; Figure 8D). Comparing the Fructose + MH-76 versus Fructose + Prazosin groups, we noticed statistically significantly enhanced vascular inflammatory symptoms in the Fructose + Prazosin treated animals, especially in venules and small veins (*p* < 0.05; Figure 8B,C) and in arterioles (*p* < 0.01; Figure 8D).

#### 2.4.3. Effect of MH-76 and Prazosin on Eosinophils Infiltration in Abdominal Adipose Tissue

**Figure 9:**
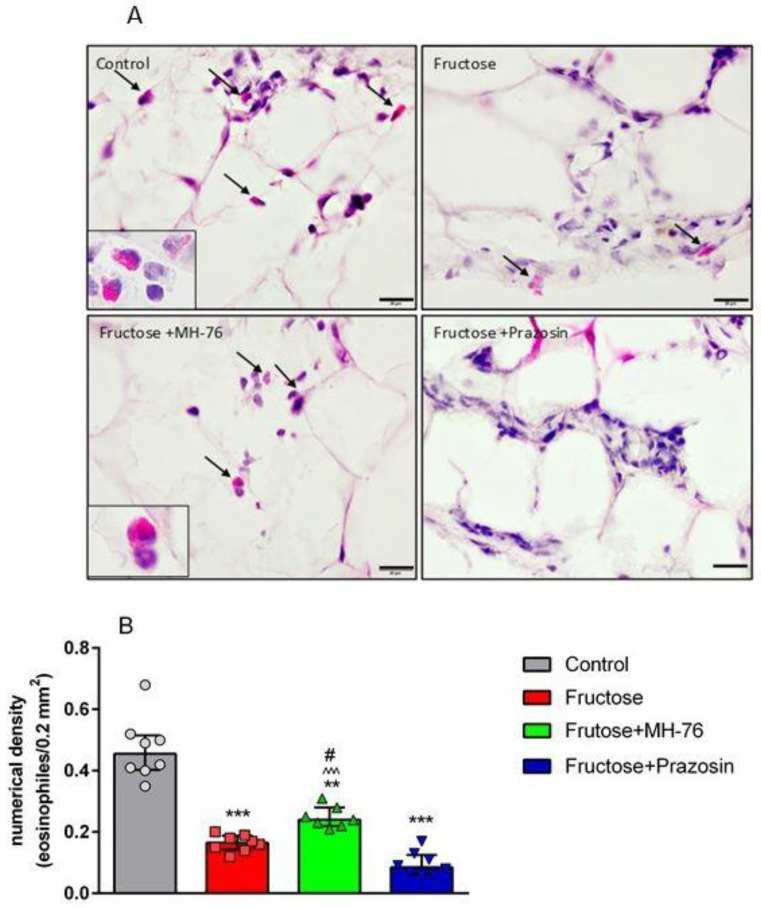
(**A**) Representative photomicrographs of abdominal adipose tissue with eosinophils (arrows and enlarged boxes) in the different groups of rats. The increase in eosinophil count in Fructose + MH-76 adipose tissue are visible. Hematoxylin and eosin stain, bar = 20 µm. (**B**) Numerical density (median +IQR) of eosinophils per 0.2 mm^2^ cross section area of abdominal adipose tissue from the Control, Fructose, Fructose + MH-76 and Fructose + Prazosin rats. ** *p* < 0.01, *** *p* < 0.001 versus Control, # *p* < 0.05 versus Fructose, ^^^ *p* < 0.001 versus Fructose + Prazosin, (Kruskal–Wallis test by ranks, post hoc Dunn test).

When analyzing the presence of inflammatory cellular infiltrates, attention was paid to eosinophils observed in the intercellular spaces mainly in adipose tissue from Control and Fructose + MH-76 rats (Figure 9A). Figure 9B shows that, in adipose tissue of all fructose-fed rats, there was statistically significantly fewer eosinophils than in animals from the Control group. However, in adipose tissue from Fructose + MH-76 rats, the density of eosinophils was markedly higher than in Fructose or Fructose + Prazosin rats.

#### 2.4.4. Results of Immunohistochemical Studies

**Figure 10:**
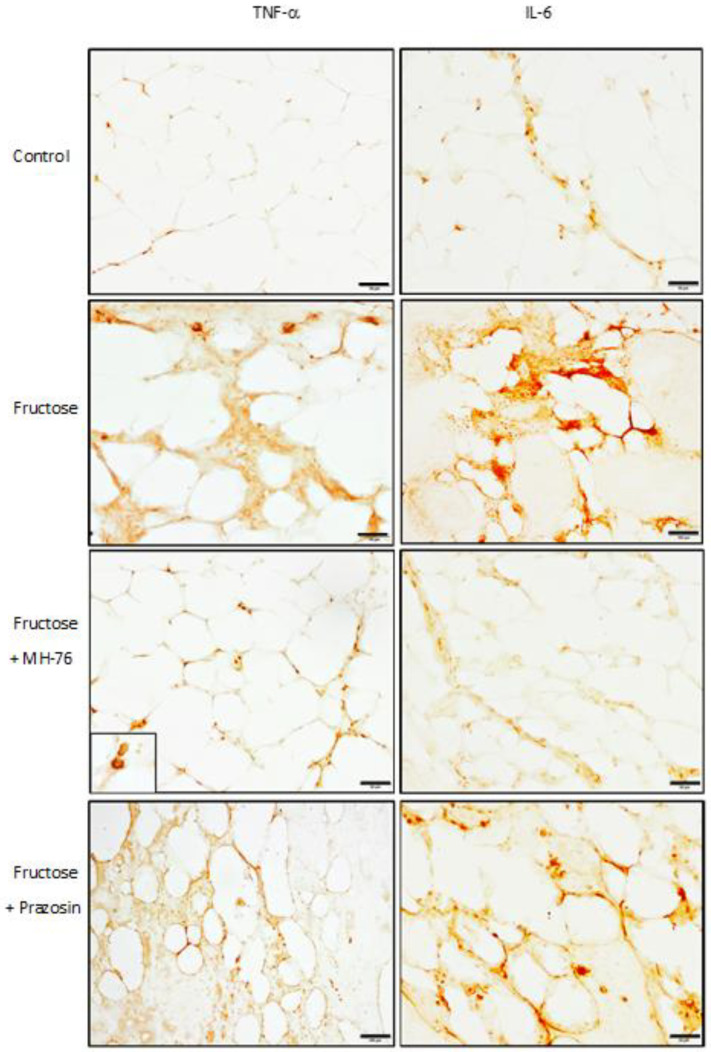
The representative immunohistochemical (IHC) photomicrographs of proinflammatory cytokines—TNF-α and Il-6 expression in the abdominal adipose tissue samples from studied groups. Both IL-6 and TNF-α expression was present in inflammatory cells (in enlarged box), in some endothelial cells and also in adipocytes and were elevated in Fructose and Fructose + Prazosin groups. These observations are similar to that obtained from histopathological analysis (inflammatory index). Bar = 100 μm.

Immunohistochemical staining showed positive immunoreaction in the proinflammatory cells, in some endothelial cells and also in adipocytes in all obtained samples. Both IL-6 and TNF-α expressions in the adipose tissue from Fructose and Fructose + Prazosin rats were elevated. Only treatment with MH-76 markedly reduced expression of those cytokines (Figure 10). This is in line with IL-6 and TNF-α concentrations determined by bead-based multiplex (IL-6, Figure 2D) and immunoenzymatic assays (TNF-α, [24]), in which the levels of both proinflammatory cytokines were statistically significantly reduced in adipose tissue from Fructose + MH-76 rats compared with Fructose and Fructose + Prazosin rats.

### 2.5. Concentrations of Prazosin and MH-76 in the Liver and Adipose Tissue

The average concentration of prazosin in the livers of rats which were administered prazosin for six weeks at the dose of 0.2 mg/kg/day equaled to 301.32 ± 171.46 ng/g, and the level of this compound in the adipose tissue of the same animals was 20.83 ± 15.42 ng/g. In the livers of rats receiving MH-76 also for 6 weeks at the dose of 5 mg/kg/day, the average MH-76 concentration was equal to 101.07 ± 36.13 ng/g, while the level observed in the adipose tissue was 1372.33 ± 237.13 ng/g. The data are summarized in Table S1.

## 3. Discussion

Increasing evidence indicates that extensive fructose consumption, obesity and metabolic syndrome are strongly associated with pro-inflammatory signaling in many tissues including adipose tissue. A high fructose diet triggers the production of inflammatory cytokines, followed by an inflammatory response in adipose tissue, manifested with morphological alterations of adipocytes. These changes further increase visceral adiposity and fat accumulation and cause impairment in insulin signaling in adipose tissue of humans or rats fed a high fructose diet [4,11,27].

MH-76 is a potent antagonist of α_1_-adrenoceptors that was confirmed in rat α_1A_-, α_1B_- and α_1D_- [22] as well as human α_1A_-adrenoceptors, and it did not show partial agonist properties. In our previous studies, we showed that MH-76, in addition to its antihypertensive effect, decreased hyperglycemia and hypertriglyceridemia in fructose-fed rats. Moreover MH-76 reduced abdominal adiposity and insulin resistance, as well as decreased TNF-α concentration and lipid peroxidation in adipose tissue. Prazosin treatment although exerted an antihypertensive effect and reduced hyperglycemia did not reduce insulin resistance or abdominal adiposity [24]. As a continuation of our previous research, in this study we looked deeper and further investigated the positive effect of MH-76 on inflamed adipose tissue in metabolic syndrome since this particular tissue is considered to be the major source of the pro-inflammatory cytokines and a key player in obesity linked disorders [28,29].

The results from our both previous and current biochemical and histopathological assays clearly indicate that excessive consumption of fructose leads to an increase in both mass and size of adipocytes as well as inflammation and endocrine dysfunction of adipose tissue. In particular, in fructose-fed rats we observed massive infiltration of different inflammatory cells, decreased adiponectine and increased leptine concentrations and increased levels of pro-inflammatory cyto/chemokines (MCP-1, IL-6, TNF-α). Treatment with MH-76 but not with prazosin proved to alleviate some of these conditions.

A major characteristic of abdominal obesity is the accumulation of fat deposition and an increase in adipocyte size. Increased fat cell size has been considered to be a marker or even a driver of metabolic disease and adipose tissue dysfunction [30]. Since treatment with MH-76 reduced abdominal adiposity, we were wondering if that effect could be associated with the reduction in the number of large adipocytes. In MH-76-treated rats, abdominal fat loss could have been associated with the reduction of the number of large adipocytes that, subsequently, caused an increase of numerical density of adipocytes. However, the mean size of adipocytes in rats from MH-76 treated and from the Fructose group was similar. Interestingly, the highest number of very large adipocytes was observed in the Fructose + Prazosin group, which correlated with high expression of pro-inflammatory cytokines. Moreover, we have observed a strong negative correlation between abdominal fat weight [24] and the numerical density of adipocytes.

In healthy adipose tissue, there are only adipocytes and very few inflammatory leukocytes. Hypertrophy of adipose tissue is usually followed by increased infiltration of immune cells and cytokine secretion [28]. Subsequently, many different leukocytes including macrophages, mast cells, lymphocytes and neutrophils accumulate in the adipose tissue, contributing to inflammation [28,29]. Indeed, histopathological evaluation of adipose tissue from fructose and fructose-fed, prazosin treated rats showed massive recruitment of inflammatory cells, fibrosis and adipocyte hypertrophy. In fact, the most severe inflammation was observed in adipose tissue from Fructose + Prazosin group. Contrary, slight inflammation involving mostly septa was seen in adipose tissue from Fructose + MH-76 rats, which suggested the anti-inflammatory effect of this compound. We have also found a significant positive correlation between abdominal fat weight and inflammatory index.

Inflammation is regulated by cytokines, which are produced and released by different inflammatory cells. In obesity, adipose tissue infiltrating leukocytes are probably the main source of locally-produced pro-inflammatory cytokines. However, adipocytes themselves also secrete them in substantial amounts. Cytokines further activate the inflammatory program in neighboring adipocytes, exacerbating inflammation [29]. Mediators derived from an expanded pool of adipocytes and tissue macrophages are likely responsible for microvascular dysfunction, which further contributes to both the initiation and propagation of the inflammatory response [31]. The microvasculature responds to these mediators by enhanced expression of the endothelial cell adhesion molecules, that leads to leukocytes peripheralization cascade, and consequently to impaired endothelial barrier function and increased vascular permeability. Such inflammatory phenotype in regard to endothelial damage symptoms, leukocyte adhesion and extravasation, as well as perivascular lymphocyte and macrophage aggregation, was shown in the Fructose group. This pathology was not only preserved, but markedly strengthened in the Fructose + Prazosin group especially in venules and smallest veins. Interestingly, Fructose + MH-76 treated animals showed a normalization of the inflammatory response. We reported previously that MH-76 but not prazosin reversed impaired endothelial vascular relaxation in fructose-fed rats [24], and that MH-76 exerted endothelial activity through activating NO/sGC/cGMP signaling pathway [23]. In addition to anti-inflammatory properties, these effects may also account for MH-76 beneficial influence on endothelial integrity in adipose tissue microvasculature found in our studies.

Several works [28,32,33] demonstrated a beneficial effect of eosinophils on the course of inflammation in adipose tissue in animal studies. It has been shown that eosinophils migrating to the septa between adipocytes are the main IL-4 producing cells. IL-4 is involved in the activation of M2 macrophages, which in turn are strongly engaged in tissue repair and reconstruction. Moreover, eosinophils influence the morphology of adipose tissue by modulating the size of adipocytes and intercellular spaces. The presence of eosinophils is therefore essential in the healing process of inflammatory cell infiltrates and also necessary for the proper morphology of adipose tissue. It has been shown previously that in obesity the quantity of adipose tissue eosinophils decreases [9,28,29,34]. Indeed, in our study we have found that the density of eosinophils was markedly lowered in adipose tissue from Fructose and Fructose + Prazosin animals, whereas the amount of eosinophils in adipose tissue of Fructose + MH-76 rats was markedly higher than in other fructose-fed animals. We have also found that the higher density of eosinophils was accompanied with a higher concentration of IL-4, the phenomenon observed only in adipose tissue of control and fructose-fed, MH-76 treated rats. Data from animal studies show that eosinophils may also exert a beneficial effect on insulin sensitivity in adipose tissue [32,33,34]. It is in line with our studies, where the number of eosinophils was associated with the healing of inflamed tissue and the reduction of insulin resistance. However, it must be pointed out that some human studies do not support a protective role of eosinophils in metabolic syndrome [35] and the relationship between eosinophils and adipose tissue requires further studies.

A vital role in the maintenance of insulin sensitivity plays adiponectin, the most abundant adipose-derived protein, that also exerts a pronounced anti-inflammatory effect [1,3,29]. Many studies reported that in abdominal adiposity caused by fructose feeding adiponectin secretion was reduced [11]. Indeed, in our studies fructose consumption caused a decrease of adiponectin concentration in adipose tissue, which was restored by treatment with both α_1_-adrenoceptors antagonists, which what may indicate that this beneficial effect is α_1_-adrenoceptor dependent.

Adipocytes are also the source of leptin, a hormone involved in controlling food intake, feeding behavior and energy homeostasis [11,28]. High blood levels of leptin play a major role in insulin resistance by inducing the production of IL-6 and TNF-α [10]. Leptin concentrations are markedly increased in obesity, including fructose feeding model, and positively correlated with adipose mass, indicating leptin resistance [11,36]. This again is in line with our studies; in adipose tissue from fructose-fed rats we found higher leptin concentration, which was reduced by treatment with MH-76 but not with prazosin. Leptin receptors are also expressed on vascular endothelial cells and their activation induce the proinflammatory phenotype resulting in an increased capacity to recruit immune cells [37]. In our studies, MH-76 but not prazosin decreased leukocyte adhesion and extravasation in adipose tissue.

Treatment with MH-76, contrary to prazosin, also reduced the levels of some proinflammatory cytokines such as TNF-α [24], MCP-1 or IL-6. TNF-α is overexpressed in adipose tissue of obese rodents and humans, which is related to increased infiltration of inflammatory cells, mainly macrophages. Their recruitment involves the synthesis of chemokines such as MCP-1 [2,29]. As a response to a high concentration of MCP-1, there is an increased infiltration of monocytes to adipose tissue, where they are differentiated into macrophages responsible for further increase in pro-inflammatory cytokines, resulting in highly inflammatory environment [28]. Lowering concentrations of TNF-α and MCP-1 in adipose tissue of fructose-fed rats suggests the strong anti-inflammatory activity of MH-76. Adipocytes and macrophages produce also IL-6, which expression is enhanced in obesity [9,11]. Indeed, increased tissue expressions of IL-6 and TNF-α were found in macrophages, in some endothelial cells and also in adipocytes; however, they were elevated only in adipose tissue from Fructose and Fructose + Prazosin animals, confirming the anti-inflammatory effect of MH-76.

At the molecular and cellular levels, several signaling pathways have been proposed to link inflammation and insulin resistance [1,2]. Typically, as insulin binds to its receptor, it stimulates autophosphorylation of tyrosine residues. Activated insulin receptor (IR) phosphorylates selective tyrosine residues on IRS, and the phosphorylated proteins are able to activate different signaling pathways. The activation of phosphatidylinositol 3-kinase (PI3K), mainly through IRS-1, is preferentially involved in the metabolic effects of insulin.

One mechanism mediating insulin resistance involves phosphorylation of serine residues in IRS-1, inducing its conformational change that in turn can lead to disruption of the interaction between IR and IRS-1 [8,34]. There are several serine phosphorylation sites on IRS-1 and their phosphorylation is stimulated by cytokines [38]. Various kinases, which can be stimulated by TNF-α and IL-6, such as Jun NH2-terminal kinase (JNK), an inhibitor of NF-κB kinase (IKK) and protein kinase C (PKC) are candidates that can mediate insulin resistance [1,39]. TNF-α is known to be involved in the disturbance of insulin/IR-initiated signals by activating JNK-1 responsible for phosphorylation of Ser307 in IRS-1 [1]. Thus, TNF-α induces phosphorylation of IRS-1 at the serine 307 residue, decreasing insulin sensitivity in adipose tissue [11,29,38,39]. This leads to progressive accumulation of IRS-1 molecules phosphorylated at Ser307, which in turn results in reduced interaction between IRS-1 and the IR or PI3K, and inhibition of insulin signaling [10,38,39]. It also has been shown that IKK-β by phosphorylating inhibitory serine residue on IRS-1 or activating NF-κB, a transcription factor, promotes further inflammatory gene expression and stimulates the expression of inflammatory mediators such as TNF-α or IL-6 [1]. Like TNF-α, also IL-6 alters insulin sensitivity and impairs insulin signaling through serine phosphorylation of IRS-1 [1,29,40]. All these factors impair insulin signal transduction through IRS and PI3K kinase pathway [28].

Consistent with these studies, we observed elevated levels of TNF-α and IL-6 as well as enhanced Ser307 phosphorylation of IRS-1 in fructose-fed rats. Treatment with MH-76 markedly reduced the phosphorylation of IRS-1 at Ser307, which may explain the reduction of insulin resistance and hyperinsulinemia observed in our previous study [24]. The observed modulation of insulin signaling by MH-76 might be attributed to both its anti-inflammatory effect and the reduction of increased levels of cytokines such as TNF-α and IL-6.

Finally, we have found the increased PAI-1 concentration in adipose tissue from fructose-fed animals, which was effectively lowered by treatment with MH-76 but not with prazosin. PAI-1, a serine protease inhibitor, is a marker of impaired fibrinolysis and atherothrombosis. PAI-1 over-expression is associated with atherosclerosis in humans, especially in patients with the metabolic syndrome [8,41]. It has been suggested that abdominal adipose tissue is the major source of plasma PAI-1, as it originates from adipose tissue in response to chronically elevated levels of TNF-α and insulin in individuals with metabolic syndrome [1,3,8].

An additional aspect, which also has to be taken into account, is that the doses of prazosin and the MH-76 compound administered to experimental animals were different (0.2 vs. 5 mg/kg b.w.). They were selected based on the affinity of the investigated compounds for α_1_-receptor (Ki = 0.1 or 2 nM for prazosin and MH-76, respectively); therefore, the tested dose of MH-76 was 25 times higher than that of prazosin. However, due to the low bioavailability of MH-76 after i.p. administration, the total exposure to this compound was only 3.7 times higher than to prazosin [24]. Thus, it seems that the compound MH-76 has more beneficial, as compared to prazosin, pharmacological properties at plasma concentrations much lower than expected. Therefore, we have directly measured the concentrations of prazosin and MH-76 in the target organs, namely liver and adipose tissue. We have determined that MH-76, preferably distributed into the abdominal fat, and its concentration in this tissue was almost 70 times higher than that of prazosin (1372.33 vs. 20.83 ng/g). Such pharmacokinetic property might also be involved in the overall beneficial effect of MH-76. On the other hand, studies performed on cells expressing human α_1_-adrenoceptor showed that the antagonistic potency of prazosin towards α_1_-adrenoceptor is two times higher compared to MH-76 (0.68 nM vs. 1.42 nM). The dose normalized concentration of MH-76 in fat tissue was ca. two times higher than prazosin (Supplementary Table S1). Therefore, with high probability, we can say that these two drugs produce a comparable degree of α_1_-adrenoceptor antagonism in adipocytes at chosen doses.

In the end, it should be emphasized that prazosin not only did not reduce but even augmented the inflammation in the adipose tissue, exacerbating the recruitment of inflammatory cells and fibrosis. Consequently, prazosin did not reduce the elevated concentrations of leptin, TNF-α, IL-6 and MCP-1, and did not improve insulin signaling. Similar results we found in our previous studies, in which in DOCA-salt hypertensive rats, treatment with prazosin, despite the strong blood-pressure-lowering activity, revealed detrimental effects on coronary and renal arteries [42]. Probably, other factors than α_1_-adrenoceptor blockade (i.e., anti-inflammatory effect together with favorable pharmacokinetic parameters) determine high efficacy of MH-76, leading to the effective reduction in inflammatory status in adipose tissue.

Our study has possible limitations; although the performed experiments showed that MH-76 is an antagonist of α_1_-adrenoceptors, we cannot exclude the possibility of biased agonism or inverse agonism in its molecular mechanism of action. Further studies are required to elucidate the exact cellular mechanisms of MH-76 pharmacological activity.

## 4. Materials and Methods

Abdominal adipose tissue was collected from fructose-fed rats with metabolic syndrome from our previous experiment. All experimental procedures were conducted in accordance with the ARRIVE guidelines and with the guidelines of the National Institutes of Health Animal Care and Use Committee and approved by the Local Ethics Committee on Animal Experimentation (resolutions no. 338/2017 and 187/2018) in Krakow, Poland [24]. Male Wistar rats (Krf: (WI) WU) weighing 190–210 g, age 7 weeks, obtained from an accredited animal house at the Faculty of Pharmacy, Jagiellonian University Medical College, Krakow, Poland, were used.

Rats were divided into 4 groups (n = 7–8) and studied for 18 weeks. The groups were as follows:

Control group. Animals received regular diet and water ad libitum for 18 weeks. After 12 weeks, this group received saline (1 mL/kg ip daily) during the last 6 weeks of the experiment.

Fructose: Animals received a regular diet and fructose was administered as 20% solution in drinking water for 18 weeks. After 12 weeks, this group received saline (1 mL/kg ip daily) during the last 6 weeks of the experiment.

Fructose + MH-76: Animals received a regular diet and fructose was administered as 20% solution in drinking water for 18 weeks. After 12 weeks, this group received MH-76 5 mg/kg/day ip during the last 6 weeks of the experiment.

Fructose + Prazosin: Animals received a regular diet and fructose was administered as 20% solution in drinking water for 18 weeks. After 12 weeks, this group received prazosin 0.2 mg/kg/day ip during the last 6 weeks of the experiment [24].

**Figure 11:**
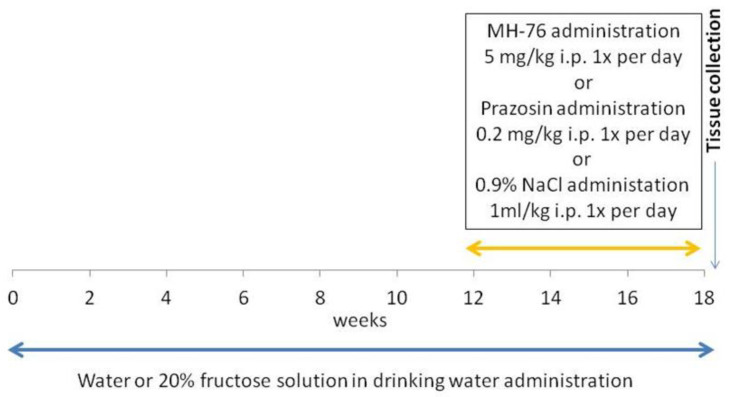
Timeline of the experiment.

Figure 11 shows timeline of the experiment.

At the end of experiment, after 16 h fasting but with free access to water, all rats were anesthetized with thiopental (75 mg/kg i.p.), decapitated and abdominal adipose tissue was dissected out and weighed by investigators unaware of the groups allocation. Abdominal adipose tissue was stored at −80 °C until assayed. Table 2 shows the number of experimental groups, final body mass and abdominal fat index at the end of experimental period [24].

### 4.1. Biochemical Assays

#### Preparation of Tissue Homogenates

The frozen abdominal adipose tissue was weighed and homogenates were prepared by homogenization of 1 g of the tissue in 4 mL of 0.1 M phosphate buffer, pH 7.4, using the IKA-ULTRA-TURRAX T8 homogenizer. Adipose tissue homogenates were next used for biochemical assays.

The concentrations of IRS-1 (E0919Ra, Bioassay Technology Laboratory), pIRS-1 (Ser307) (BC-ER140997, Biocodon Technologies) and IL-4 (E0133Ra, Bioassay Technology Laboratory) in adipose tissue were determined by the ELISA method with the use of commercially available kits. The concentrations of adiponectine, leptine, MCP-1, PAI-1 and IL-6 were determined using commercially available MilliplexTM MAP Kit (RADPCMAG-82K, Millipore, Billerica, MA, USA) according to the manufacturer’s protocol. The quantitative analysis was performed using MAGPIX Luminex analyzer with xPONENT software (Luminex Corporation, Austin, TX, USA).

Levels of analytes were calculated based on the standard curves using spline curve-fitting method and were expressed in pg/mL or ng/mL of homogenate.

### 4.2. Histopathological Analysis

#### 4.2.1. Quantitative Evaluation of Adipose Tissue

The abdominal adipose tissue samples were fixed in 4% formaldehyde and embedded in paraffin. Sections from each sample were cut (5 μm) and stained using standard staining protocols for hematoxylin and eosin (H&E) and Masson’s trichrome. The histopathological studies were performed in six images per section under 100×, 40× and 20× magnification by a pathologist in blinded randomized sections of the tissues. Images were captured with a light microscope (Olympus BX41, Japan) and color camera (Olympus UC90, Olympus). The digital images were then analyzed using computerized imaging CellSensDimension (Olympus) software. The mean adipocyte size, the size distribution of the cell populations and numerical density (adipocytes/1 mm^2^ cross section area) were measured. Adipocyte size was determined by microscopic measurement of cell diameter. About 200 adipocytes per case were measured. The numerical density of eosinophils per 0.2 mm^2^ cross section area was also analyzed. The inflammatory reaction in adipose tissue was classified in terms of the presence of infiltrated immune cells, fibrosis and widening of the interstitial space and was graded on a semiquantitative 4-point scale ranging from 0 to 3+. The mean calculated from independently assessed values was presented as an inflammatory index. An analogous semi-quantitative scale (0—minimal, 1—mild, 2—moderate, 3—marked), and the averaged index was used to examine the inflammation in the microcirculation of the adipose tissue, covering capillaries, arterioles and small arteries, venules and small veins, including vascular endothelial damage, leukocyte recruitment, adhesion and extravasation as well as perivascular lymphocyte/macrophage aggregation. Only microvasculature in the parallel alignment of descending arterioles and ascending collecting venules were taken into account to ensure vessels identification.

#### 4.2.2. Immunohistochemistry

The same paraffin slides of abdominal adipose tissue were immunohistochemically stained for TNF-α and IL-6 using the labelled streptavidin-biotinylated peroxidase system (IHC Select Immunoperoxidase Secondary Detection System, MILLIPORE, Burlington, MA, USA). Deparaffinization, rehydration and antigen retrieval were performed by placing slides in target retrieval solution (Declere, Cell Marque, Rocklin, CA, USA) under pressure cooker boiling for 30 min. After washing in TBS (Tris-buffered saline), endogenous peroxidase activity was blocked by incubating the sections in 3% H_2_O_2_/methanol for 15 min. Non-specific antibody binding sites were blocked with normal goat serum (MILLIPORE, Burlington, MA, USA) for 10 min at room temperature. Sections were incubated in a moist chamber at 4 °C overnight and then for 1 h at room temperature with 0.125 μg/mL of a mouse anti-rat monoclonal IL-6 antibody (ab9324, Abcam, Cambridge, UK), or with 1 μg/mL of a rabbit anti-rat polyclonal TNF-α antibody (ab6671, Abcam, Cambridge, UK). After several washes in TBS, the sections were incubated in a biotinylated secondary IgG and then with streptavidin-biotin horseradish peroxidase complex for 10 min at room temperature in accordance with the instructions from the producers. The binding of primary antibody was visualized using 3,3′-diaminobenzidine (DAB, MILLIPORE, Burlington, MA, USA) for 6 min. The slides were dehydrated, cleared with xylene and mounted in DPX. For each antibody tested, a negative control was performed by omitting the primary antibody. Histological slides with the expression of TNF-α and Il-6 were examined under the optical microscope connected to a digital camera under 40× magnification.

### 4.3. Tissue Distribution Studies

Concentrations of prazosin and MH-76 in adipose tissue and liver homogenates were determined using the LC-MS/MS method, validated according to the FDA guidelines [43]. Accuracy (percent of recovery) was evaluated as [mean found concentration/theoretical concentration] × 100. Precision was given by the percent relative standard deviations (RSD, CV%). The criteria for acceptability of the data included accuracy within ±15% deviation from the nominal values and a precision within 15% RSD except for LLOQ, where it should not exceed ±20% of CV. Due to the small number of samples analyzed, no stability studies were performed. Liver samples were weighted and homogenized in water at the ratio of 1:4 (w:v). After protein precipitation with acetonitrile (ACN) and centrifugation, the supernatant (10 µL) was injected onto a LC-MS/MS system. Adipose tissue samples were homogenized in acetonitrile and centrifuged, then the supernatant was diluted 100 times with ACN and 10 µL was injected onto a LC-MS/MS system. Chromatography was performed on the XBridge C18 5 µm 3 × 100 mm (Waters, Ireland) analytical column with gradient elution using a mobile phase containing ACN and water with 0.1% of formic acid. Chromatographic system Exion LC AC (AB Sciex, Vaughan, OT, Canada) was connected to the QTRAP 4500 (AB Sciex, Vaughan, OT, Canada) mass spectrometer operated in the selected reactions monitoring mode (SRM), monitoring the transition of the protonated molecular ions to their specific fragments. Two pairs were used for each compound (for MH-76 = 355/205 m/z and 355/136 m/z; for prazosin = 384/247 m/z and 384/138 m/z). Data acquisition and processing were accomplished by the Analyst version 1.7 software.

### 4.4. Drugs and Chemicals

Prazosin (prazosin hydrochloride) was purchased from Tocris, UK. Compound MH-76 was synthesized in the Department of Bioorganic Chemistry, Chair of Organic Chemistry, Pharmaceutical Faculty, Jagiellonian University [44], Figure 1. Melting point of MH-76 was measured on Büchi SMP-560 apparatus (Büchi Labortechnik AG, Switzerland) and was given as uncorrected. The purity of MH-76 was estimated by LCMS system, that consisted of Waters Acquity UPLC coupled to Waters TQD mass spectrometer (Waters, Milford, MA, USA) with electrospray ionization mode, ESI-tandem quadrupole. Acquity BEH C18, 1.7 µm, 2.1 × 100 mm column was used. M.p. 214–216; the exact mass of MH-76 base—354,23. LC-MS [M+H]+ m/z—355.045, 100% purity (Figures S1 and S2).

### 4.5. In Vitro Functional Bioassays at Cells Transfected with Human α_1A_-Adrenoceptor (Aequorin and Luminescence-Based Intracellular Calcium Assay)

Intrinsic activity assay was performed using an Aequoscreen assay according to the manufacturer of the ready to use cells with stable expression of the α_1A_-adrenoceptor (Perkin Elmer). The Aequoscreen technology uses the recombinant cell lines with stable co-expression of apoaequorin and a GPCR as a system to detect activation of the receptor, following addition of an agonist, via the measurement of light emission. For measurement, cells (frozen, ready to use) were thawed and resuspended in 10 mL of assay buffer containing 5 μM of coelenterazine h. Cells suspension was placed in a 10 mL Falcon tube, fixed onto a rotating wheel and incubated for overnight at RT° in the dark. Then cells were diluted with Assay Buffer to 2000 cells/50 µL. Agonistic ligands 2 times concentrated (50 μL/well), diluted in Assay Buffer, were prepared in 1/2 white polystyrene area plates, and the cell suspension was dispensed on the ligands in 50 μL volume using the injector. The light emission was recorded for 20 s. Cells with antagonist were incubated for 15 min at RT°. Then, 50 µL of agonist (3 times of EC_80_ final concentration—phenylephrine) was injected onto the mix of cells and antagonist and the light emission was recorded for 20 s.

### 4.6. Statistical Analysis

Results of this exploratory study are expressed as means ± Δ/2, where Δ is a width of the 95% confidence interval (CI). Statistically significant differences between groups were calculated using one-way ANOVA and the post-hoc Bonferroni multiple comparison test with all possible pairwise comparisons. For histopathological studies, results are expressed by median and inter-quartile range (IQR). For these data, the comparisons among groups were performed using Kruskal–Wallis test by ranks followed by Dunn’s post-hoc test with all pairwise comparisons. The Pearson correlation was used to assess the correlation values between the selected parameters. Differences were considered significant at *p* < 0.05.

## 5. Conclusions

In conclusion, our study showed that treatment with MH-76 reduced inflammation in adipose tissue from rats with developed metabolic syndrome, which may indicate its anti-inflammatory effect. MH-76 may improve insulin signaling by reducing the pro-inflammatory cytokine production and inhibiting the inflammatory cells recruitment in adipose tissue. In contrast, in adipose tissue from animals treated with prazosin, the inflammation was more severe, even compared to the adipose tissue from the no-treatment group.

## Figures and Tables

**Table 1:** Functional activity results for MH-76 and prazosin. Antagonist potency towards α_1A_-adrenergic receptors, expressed as half maximal inhibitory concentration-IC_50_ (nM), and and negative log of the antagonist dissociation constant-pK_B_ with SEM.

Compound	IC_50_ ± SEM (nM)	pK_B_ ± SEM
**MH-76**	1.42 ± 0.46	8.938 ± 0.112
**prazosin**	0.68 ± 0.10	9.301 ± 0.076

**Table 2:** Physiological parameters of Control, Fructose-, Fructose + MH-76- and Fructose + prazosin-treated rats at the end of the experimental period. The presented data were already published in [24]. Values are presented as mean ± Δ/2, where Δ is a width of the 95% CI.

Group	n	Final Body Weight (g)	Abdominal Fat Weight (mg/g Body wt)
**Control**	8	483.6 ± 19.2	41.11 ± 11.94
**Fructose**	8	517.8 ± 25.1 *	58.88 ± 15.09 ***
**Fructose + MH-76**	7	469.9 ± 25.9 ^## ^^^	36.21 ± 16.69 ^^^^ ###^
**Fructose + prazosin**	8	530.4 ± 25.8 *	57.67 ± 21.33 ***

* *p* < 0.05, *** *p* < 0.001 vs. Control, ## *p* < 0.01, ### *p* < 0.001 vs. Fructose, ^^ *p* < 0.01, ^^^ *p* < 0.001 vs. Fructose + Prazosin, one-way ANOVA, post hoc Bonferroni multiple comparison test.

## Data Availability

The data presented in this study are available on request from the corresponding author.

## Supplementary Materials

The following are available online at https://www.mdpi.com/article/10.3390/ph14050477/s1, Figure S1: Total absorbance chromatogram (TAC). Figure S2: MS detection in full scan mode with positive ionization. Table S1: Concentrations of MH-76 and prazosin in the liver and adipose tissue of rats at the end of the experiment. Values are presented as mean ± SD, (n = 7–8).
