# Supplementary material for: MH-76, a Novel Non-Quinazoline α1-Adrenoceptor Antagonist, but Not Prazosin Reduces Inflammation and Improves Insulin Signaling in Adipose Tissue of Fructose-Fed Rats
Source: Pharmaceuticals (Basel). 2021 May 18;14(5):477. doi: 10.3390/ph14050477 (PMC8157569; doi:10.3390/ph14050477)
Supplement: Supplementary file 1 [file pharmaceuticals-14-00477-s001.zip › pharmaceuticals-1182246-supplementary.pdf]

pharmaceuticals MDPI

Table S1. Concentrations of MH-76 and prazosin in the liver and adipose tissue of rats at the end of experiment. Values are presented as mean ±SD, (n=7-8).

Compound	Prazosin	MH-76
**Dose administered**	0.2 mg/kg/day i.p. for 6 weeks	5 mg/kg/day i.p. for 6 weeks
**Concentration in the liver at the end of experiment**	301.32±171.46 ng/g	101.07±36.13 ng/g
	**dose normalized values**	
	1506.6±857.3 ng/g/dose	20.21±7.22 ng/g/dose
**Concentration in the adipose tissue at the end of experiment**	20.83±15.42 ng/g	1372.33±237.13 ng/g
	**dose normalized values**	
	104.15±77.1 ng/g/dose	274.4±47.42 ng/g/dose

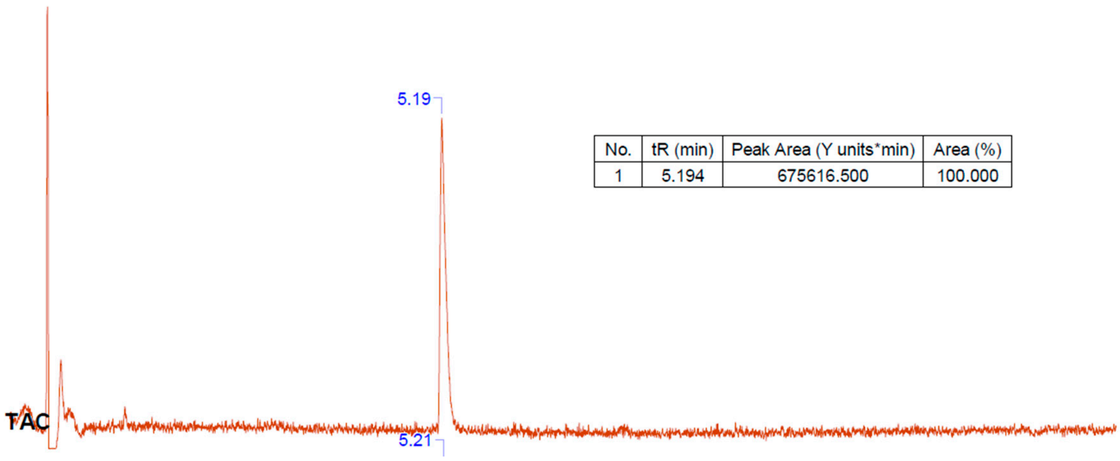

Figure S1. Total absorbance chromatogram (TAC).

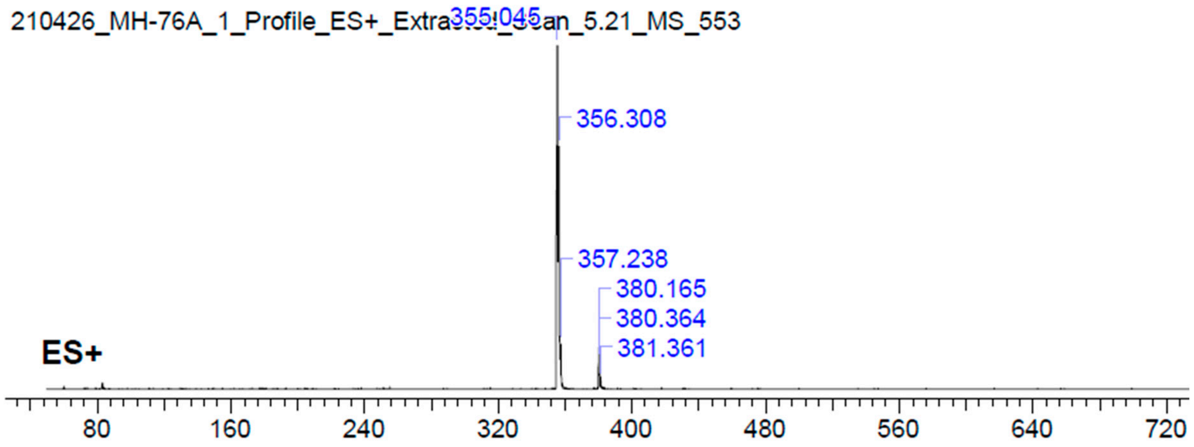

Figure S2. MS detection in full scan mode with positive ionization.
